# Supplementary material for: A scholarly network of AI research with an information science focus: Global North and Global South perspectives
Source: PLoS One. 2022 Apr 15;17(4):e0266565. doi: 10.1371/journal.pone.0266565 (PMC9012391; doi:10.1371/journal.pone.0266565)
Supplement: S1 Appendix — (DOCX) [file pone.0266565.s002.docx]

**Appendix. The query of the search**

| # | Query* | Result |
| --- | --- | --- |
| 1 | TI= (“artificial intelligence” or “intelligent agent” or “machine learning” or “expert system” or “chatbot” or “data mining” or “intelligent machine” or “big data” or “smart machine”) | 460 |
| 2 | TI= ("case-based reasoning") | 6 |
| 3 | TI= ("computer vision") | 5 |
| 4 | TI= ("cognitive computing") | 2 |
| 5 | TI= ("cognitive science") | 4 |
| 6 | TI= ("data science") | 20 |
| 7 | TI= ("fuzzy linguistic modeling") | 0 |
| 8 | TI= ("fuzzy logic") | 6 |
| 9 | TI= ("genetic algorithm") | 14 |
| 10 | TI= ("image recognition") | 1 |
| 11 | TI= ("k-means") | 5 |
| 12 | TI= ("knowledge-based system") | 1 |
| 13 | TI= ("logic programming ") | 1 |
| 14 | TI= ("machine vision") | 0 |
| 15 | TI= ("natural language processing ") | 38 |
| 16 | TI= ("neural network") | 44 |
| 17 | TI= ("pattern recognition") | 5 |
| 18 | TI= ("recommendation system") | 10 |
| 19 | TI= ("recommender system ") | 15 |
| 20 | TI= ("semantic network") | 10 |
| 21 | TI= ("speech recognition") | 5 |
| 22 | TI= ("support vector machine") | 8 |
| 23 | TI= ("text mining") | 62 |
| 24 | Include all the searches without redundant using Boolean function ‘OR’, resulting 677 documents. In line with the main interest of this research, articles published in library-focused journals (n= 43) and articles with less than average citation times (n=485) were excluded. | 149 |
